# The Autophagy-Related Marker LC3 Can Predict Prognosis in Human Hepatocellular Carcinoma

**DOI:** 10.1371/journal.pone.0081540

**Published:** 2013-11-25

**Authors:** Yoo Jin Lee, Yu Jin Ha, Yu Na Kang, Koo Jeong Kang, Jae Seok Hwang, Woo Jin Chung, Kwang Bum Cho, Kyung Sik Park, Eun Soo Kim, Hye-Young Seo, Mi-Kyung Kim, Keun-Gyu Park, Byoung Kuk Jang

**Affiliations:** 1 Department of Internal Medicine, Keimyung University School of Medicine, Daegu, Republic of Korea; 2 Department of Pathology, Keimyung University School of Medicine, Daegu, Republic of Korea; 3 Department of Surgery, Keimyung University School of Medicine, Daegu, Republic of Korea; 4 Department of Internal Medicine, Kyungpook National University School of Medicine, Daegu, Republic of Korea; Xiangya Hospital of Central South University, China

## Abstract

**Background:**

Defects of autophagy and endoplasmic reticulum (ER) stress are related to many diseases and tumors. However, only a few studies have examined hepatocellular carcinoma (HCC) as related to these processes. Therefore, in this study, we investigated the expression and extent of autophagy and ER stress-related markers in HCC and their influence on clinical characteristics and prognosis for each protein.

**Methodology:**

The expression of autophagy-related markers (LC3 and Beclin-1) and ER stress-related markers (GRP78 and CHOP) was analyzed by immunohistochemistry on tissues from completely resected specimens of 190 HCC patients. Their influence on clinicopathologic features and prognosis were evaluated using the chi-square test and Kaplan-Meier analysis. Correlations of each protein were determined by Spearman's correlation analysis.

**Principal Findings:**

LC3 expression was not correlated with TNM, BCLC stage, or Edmonson-Steiner grading, whereas it was correlated with longer overall survival (OS) (*p* = 0.039) and tended to be related with longer time to recurrence (TTR) (*p*=0.068) although it did not show statistical significance. Multivariate analysis indicated that LC3 expression was a significantly independent prognostic factor of OS (HR, 0.42; 95% CI, 0.22-0.80; *p*-value=0.009) and TTR (HR, 0.54; 95% CI, 0.33–0.90; *p*=0.017). Expression of LC3 in advanced stages of TNM (III) (*p*=0.045) and Edmonson-Steiner Grades (III and IV) (*p*=0.043) was correlated with longer survival, but not in the early stages. A positive correlation was not observed between the expression of autophagy-related markers and ER stress-related markers.

**Conclusion:**

Our results suggest that the expression and extent of LC3 might be a strong prognostic factor of HCC, especially in patients with surgical resection.

## Introduction

Hepatocellular carcinoma (HCC) is the sixth most common malignancy and the third most common cause of cancer-related deaths worldwide [1]. One reason for the poor prognosis of HCC is that most therapies are effective only if HCC is diagnosed in its early stages [2]. Although there are several prognostic factors for patients with HCC, their roles remain unclear. Therefore, there is a need for better predictors of HCC prognosis that are not influenced by other clinical parameters. 

Autophagy is an intracellular catabolic process through which cells degrade and recycle their organelles or cell components using lysosomes. Autophagy redistributes nutrients from unnecessary to essential processes and maintains homeostasis in a nutrient-deprived state [3]. Several proteins are related to the autophagy pathway; the Beclin-1 and microtubule associated protein 1 light chain 3 (LC3) stimulatory pathways are key. Defects in the autophagy system lead to tumorigenesis in various organs. 

The endoplasmic reticulum (ER) is an essential intracellular organ that plays an important role in producing activated proteins, regulating calcium concentrations, and providing a site for lipid and sterol biosynthesis [4]. In harmful physiological or pathological circumstances, ER stress occurs and cells use a defensive mechanism to overcome this phenomenon, known as the unfolded protein response (UPR) [5]. The UPR starts by activating glucose-regulated protein 78 (GRP78)/binding immunoglobulin protein (BiP), which is overexpressed in lung, breast, and colon cancers, and the degree of expression of which is related to the prognosis [6-8]. The transcription factor C/EBP homologous protein (CHOP)/growth arrest and DNA damage 153 (GADD 153) is a proapoptotic pathway that originates from the stressed ER, and the expression of CHOP is related to longer survival in melanoma and breast cancer and shorter survival in non-small-cell lung cancer [9-11]. 

### There are reports that the UPR activates the autophagic pathway to remove misfolded

proteins that are not removed by ER-stress-associated degradation [12,13]. In this way, autophagy seems to play an important role in cell survival after ER stress [13]. Studies have shown that many diseases are related to autophagy defects and ER stress, which act on tumorigenesis and cancer invasion [10,14]. In HCC, however, the relationships between autophagy defects or ER stress expression and clinicopathological characteristics and prognosis remain largely unknown. Therefore, we investigated the expression of markers of autophagy and ER stress using immunohistochemistry in a sample of patients with resected HCC. We also evaluated the influence of these markers on the clinicopathological features and prognosis.

## Materials and Methods

### Patients and Study Design

 We retrospectively analyzed 190 patients with HCC that was confirmed histologically after surgical resection at Keimyung University Dongsan Hospital in Daegu, Korea, from 2001 to

2011. We included patients who had undergone hepatectomies and found to have complete resections retrospectively, without prior treatment such as transarterial chemoembolization or local ablation therapy. Completed hepatic resection was defined by the absence of microscopic tumor invasion of the resection margins [15]. Variables considered included the demographic data, the cause of HCC(e.g., alcohol, hepatitis B virus (HBV), hepatitis C virus (HCV), and HBV combined with HCV), concomitant liver cirrhosis, Child–Pugh (C–P) class, and tumor size of the resected specimens. All patients were staged using the American Joint Committee on Cancer (AJCC 2010, 7th edition) TNM staging system [16] and Barcelona Clinic Liver Cancer (BCLC) staging system for HCC [17]. Histological staging followed the Edmondson–Steiner system [18]. The C–P scoring system [19] was used to evaluate the functional status of the liver. Survival and mortality were investigated by examining the patients’ final medical records. Overall survival (OS) was counted from the date of diagnosis to the date of death or the last follow-up. Time to recurrence (TTR) was defined as the period from the date of operation to the date of recurrence.

### Immunohistochemical Staining and Scoring

 The formalin-fixed, paraffin-embedded tissue samples were reviewed to produce tissue microarray (TMA) blocks. Two 5-mm-diameter cores of representative areas were selected from each tumor sample, and these were manually embedded in new paraffin blocks using skin biopsy needles. 

 The expression of autophagy-related markers (LC3 and Beclin-1) and ER-stress-related markers (GRP78 (BiP) and CHOP) was analyzed by immunohistochemistry using tissues from completely resected specimens of HCC. Sections (4 μm) from the TMAs were placed on slides and then dewaxed and rehydrated in a graded alcohol series. Immunohistochemical (IHC) staining was performed using an automated system (Autostainer 360, Lab Vision, Fremont, CA). The primary antibodies used in this investigation were LC3 (Novus Biologicals, Littleton, CO), Beclin-1 (Epitomics, San Diego, CA), GRP78 (Cell Signaling Technology, Beverley, MA), and CHOP (Novus Biologicals, Littleton, CO). IHC expression was evaluated using the proportion score described as the estimated fraction of positively stained tumor cells (0, none; 1, <10%; 2, 10–50%; 3, >50%), and the intensity score representing the estimated staining intensity (0, no staining; 1, weak; 2, moderate; 3, strong), and calculating the total immunostaining score equaling the proportion score multiplied by the intensity score. The total score ranged from 0 to 9, with 0–4 was defined as being negative and 6–9 as positive [6,14,20].

**Figure 1:**
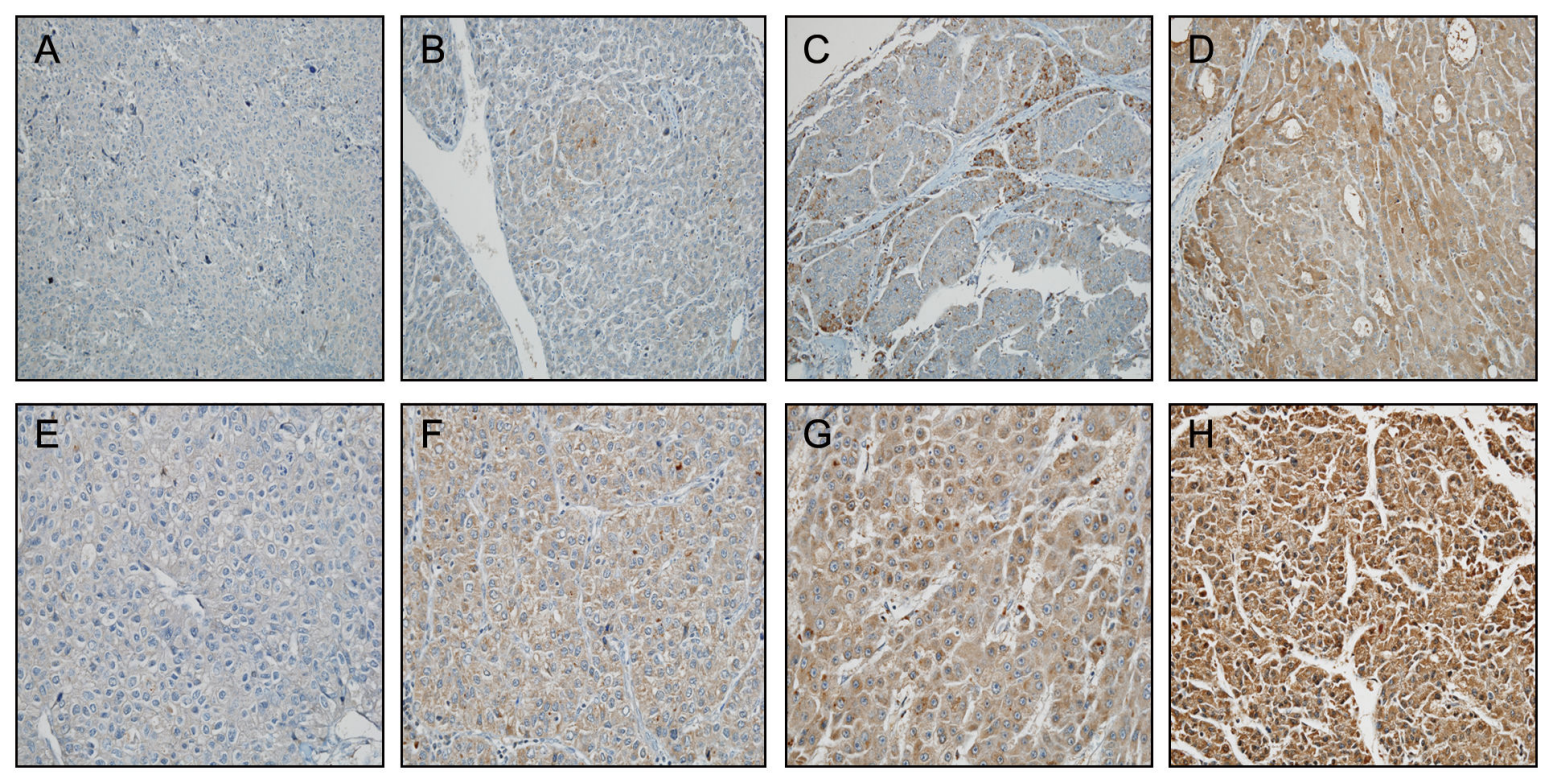
LC3 expression by immunohistochemistry in resected hepatocellular carcinoma (HCC). Representative images of areas according to the proportion of positive cells (A–D) and intensity of staining (E–H). (A) none, (B) < 10%, (C) 10–50%, (D) > 50%; and staining (E) absent, (F) weak, (G) moderate, (H) strong. (LC3 stain, ×100) (upper panel, X 200; lower panel, X 400).

Three patterns of LC3 reactivity were identified: (1) diffuse cytoplasmic, (2) cytoplasmic/juxtanuclear (perinuclear), and (3) a “stone-like” pattern in the form of dense, rounded, amorphous structures, averaging 5 μm, typically enclosed within LC3-positive cytoplasmic vacuoles. Figure 1 shows images of IHC staining for LC3. Images of IHC stainings for other markers were also evaluated (Figure S1, S2 and S3). All slides were evaluated independently three times by one senior pathologist blinded to the patients’ clinical information and follow-up data.

### Statistical Analysis

Data management and statistical analyses were performed using SPSS ver. 18.0 (SPSS, Chicago, IL, USA). Associations between each marker and clinical characteristics were evaluated using Pearson’s χ^2^ test and Fisher’s exact test. Correlation coefficients between the expression of each marker were determined using Spearman’s correlation analysis. Univariate analyses for OS and TTR for each marker were evaluated using Kaplan–Meier survival curves, and multivariate analyses were evaluated using Cox’s proportional hazard regression analysis. A *p*-value < 0.05 was considered to indicate statistical significance.

### Ethics statement

This study was approved by the Institutional Review Board of Keimyung University Dongsan Hospital (IRB No.11-256). Informed consent was waived by the board.

## Results

### LC3, Beclin-1, GRP78, and CHOP expression in HCC and their association with clinical characteristics

The baseline characteristics of the 190 patients and the associations of each marker with various clinical parameters are shown in Table 1. Expression of LC3, Beclin-1, GRP78, and CHOP was positive in 21.1% (40/190), 5.8% (11/190), 31.6% (60/190), and 3.3% (6/190) of the HCC specimens, respectively. The mean patient age was 55.7 (range 29–76) years with a male predominance (83.2%). Nearly half of the patients (97, 51.1%) were older than 55 years. The most frequent cause of HCC was the presence of HBV infection (137, 72.1%). Liver cirrhosis was present in 96 (50.5%) patients. A serum α-fetoprotein (AFP) level >200ng/mL was found in 56 (29.5%) patients. Of the patients, 188 (98.9%) were C–P class A, while only two (1.1%) were C–P class B. Fewer patients had BCLC stages 0 and A (74, 38.9%) than BCLC stages B and C (116, 61.1%). More patients had TNM stages I and II (110, 57.9%) than TNM stage III (80, 42.1%). Over half of the tumors (114, 60%) were less than 5 cm. The percentage of Edmondson–Steiner grades I and II and grades III and IV were 18.9% and 81.1%, respectively.

**Table 1:** Correlation between LC3, Beclin-1, GRP78, CHOP expression and clinicopathological characteristics of patients with HCC resection.

		**LC3**			**Beclin-1**			**GRP78**			**CHOP**		
**Characteristics**	total	Negative	Positive	*p*-value	Negative	Positive	*p*-value	Negative	Positive	*p*-value	Negative	Positive	*p*-value
All cases	190	150(78.9%)	40(21.1%)		179(94.2%)	11(5.8%)		130(68.4%)	60(31.6%)		184(96.8%)	6(3.3%)	
Male gender	158(83.2%)	126(79.7%)	32(20.3%)	0.548	150(94.9%)	8(5.1%)	0.400	108(68.4%)	50(31.6%)	0.965	155(98.1%)	3(1.9%)	0.061
Age > 55	97 (51.1%)	74(76.3%)	23(23.7%)	0.359	88(90.7%)	9(9.3%)	0.058	66(68.0%)	31(32.0%)	0.908	94(96.9%)	3(3.1%)	0.979
HCC cause				0.082			0.373			0.054			0.186
Alcohol	14 (7.4%)	7(50.0%)	7(50.0%)		12(85.7%)	2(14.3%)		7(50.0%)	7(50.0%)		14(100.0%)	0(0%)	
HBV**^*1*^**	137(72.1%)	110(80.3%)	27(19.7%)		130(94.9%)	7(5.1%)		90(66.7%)	47(34.3%)		133(97.1%)	4(2.9%)	
HCV**^*2*^**	14 (7.4%)	11(78.6%)	3(21.4%)		14(100.0%)	0(0%)		13(92.9%)	1(7.1%)		13(92.9%)	1(7.1%)	
Etc	20(10.5%)	18(90.0%)	2(10.0%)		18(90.0%)	2(10.0%)		15(75.0%)	5(25.0%)		20(100%)	0(0%)	
HBV+HCV^3^	5(2.6%)	4(80.0%)	1(20.0%)		5(100.0%)	0(0%)		5(100%)	0(0%)		4(80.0%)	1(20.0%)	
Liver cirrhosis	96 (50.5%)	76(79.2%)	20(20.8%)	0.940	88(91.7%)	8(8.3%)	0.213	64(66.7%)	32(33.3%)	0.599	92(95.8%)	4(4.2%)	0.186
AFP^4^ > 200ng/mL	56 (29.5%)	47(83.9%)	9(16.1%)	0.276	55(98.2%)	1(1.8%)	0.179	41(73.2%)	15(26.8%)	0.358	55(98.2%)	1(1.8%)	0.672
C-P class B	2 (1.1%)	2(100%)	0(0%)	0.666	2(100.0%)	0(0%)	1.000	2(100.0%)	0(0%)	1.000	2(100.0%)	0(0%)	1.000
BCLC stage B,C	116(61.1%)	91(78.4%)	25(21.6%)	0.833	107(92.2%)	9(7.8%)	0.207	79(68.1%)	37(31.9%)	0.906	70(94.6%)	4(5.4%)	0.210
TNM stage III	80(42.1%)	63 (78.8%)	17(21.3%)	0.955	74(92.5%)	6(7.5%)	0.531	58(72.5%)	22(27.5%)	0.302	79(98.8%)	1(1.2%)	0.247
Tumor size≤5cm	114(60%)	90(78.9%)	24(21.1%)	1.000	107(93.9%)	7(6.1%)	1.000	77(67.5%)	37(32.5%)	0.750	109(95.6%)	5(4.4%)	0.405
Edmondson-Steiner Grades III, IV	154(81.1%)	125(81.2%)	29(18.8%)	0.120	146(94.8%)	8(5.2%)	0.439	106(68.8%)	48(31.2%)	0.801	150(97.4%)	4(2.6%)	0.318
AST >50	55(28.9%)	41(74.5%)	14(25.5%)	0.342	50(90.9%)	5(9.1%)	0.302	35(63.6%)	20(36.4%)	0.365	50(90.9%)	5(9.1%)	0.008
ALT >50	52(27.4%)	41(78.8%)	11(21.2%)	0.983	48(92.3%)	4(7.7%)	0.496	34(65.4%)	18(34.6%)	0.580	49(94.2%)	3(5.8%)	0.348

**^1^**hepatitis B virus, **^2^**hepatitic C virus, **^3^** hepatitic B combined with hepatitis C virus, **^4^** alpha-fetoprotein

 There were no significant differences between autophagy and ER stress marker levels according to age, gender, the cause of HCC, liver cirrhosis, AFP, AST, C–P class, TNM stage,

BCLC stage, tumor size, and Edmondson–Steiner grade, with the exception of CHOP expression, which had a higher proportion of AST > 50 IU/mL compared to the lack of CHOP expression (*p*=0.008).

### Correlation between the Expression of ER Stress-Related Proteins and Autophagy-Related Proteins

Beclin-1 expression was weakly, but significantly, correlated with GRP78 (Spearman *r*=0.233, *p*=0.001), CHOP (Spearman *r*=0.187, *p*=0.009), and LC3 (Spearman *r*=0.162, *p*=0.023) expression. However, the correlation coefficients were too low to be meaningful, which suggests these markers were less affected by each other. No significant positive correlation was observed between the expression of CHOP with GRP78 (Spearman *r*=0.015, *p*=0.836), LC3 with GRP78 (Spearman *r*=0.106, *p*=0.145), and CHOP with LC3 (Spearman *r*=0.084, *p*=0.247). Overall, the Spearman’s correlation analysis found no significant correlation between autophagy and each ER stress marker.

### The relationship of markers of autophagy and ER stress with overall survival in HCC

**Figure 2:**
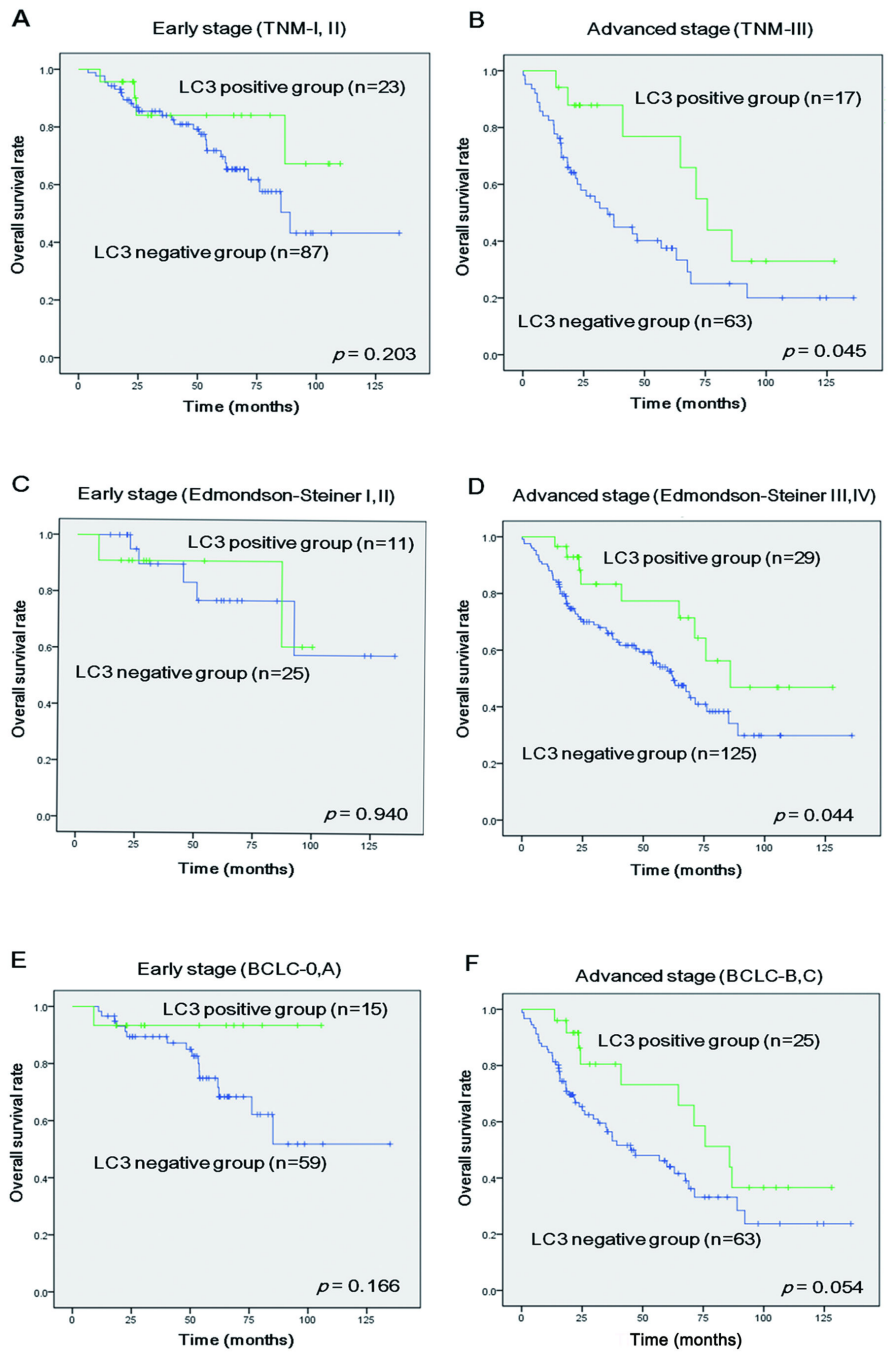
Kaplan–Meier survival analysis of LC3 expression in hepatocellular carcinoma (HCC) patients according to stage. LC3 expression in HCC patients with (A) early (I,II) and (B) advanced TNM stage (III), (C) early (I, II) and (D) advanced Edmondson-Steiner Grades (III, IV), (E) early (0,A) and (F) advanced BCLC stages (B,C). The *p* - value calculated by the log-rank test is indicated.

 During the median follow-up period of 31.4±1.4l (range 0.2–136.0) months, 114 (60.0%) patients were alive. In the univariate analyses, small tumor size (*p*<0.001), BCLC stages 0 and A (*p*<0.001), TNM stages I and II (*p*<0.001), Edmondson–Steiner Grades I and II (*p*=0.008), Child–Pugh class A (*p*=0.028), positive LC3 expression (*p*=0.039), and low serum AST (*p*=0.016) were significantly correlated with longer OS, retrospectively (Table 2). Expression of other autophagy and ER stree-related markers including Beclin-1, GRP78, and CHOP were not associated with OS in HCC (Table S1). Finally, only the following parameters were independent predictors of OS in the multivariate analysis: BCLC stages B and C (HR, 2.14; 95% CI, 1.06- 4.32; *p*=0.035), Edmondson–Steiner Grades III and IV (HR, 3.42; 95% CI, 1.55–7.57; *p*=0.002), and positive LC3 expression (HR, 0.42: 95% CI, 0.22–0.80; *p*=0.009) (Table 3). To further examine the clinical correlations, we analyzed LC3 expression according to HCC stage. LC3 expression in advanced TNM (stage III) (*p*=0.045, Figure 2(A,B)) and Edmondson- Steiner grades III and IV (*p*=0.044, Figure 2(C,D)) was correlated with longer OS, but not in the early stages. In advanced BCLC (stages B and C), LC3 expression tended to be correlated with longer OS (*p*=0.054, Figure 2(E,F)).

**Table 2:** Univariate analyses for overall survival of patients with HCC resection.

**Variables**	**No. of Patients (n=190)**	**Mean survival (Mo)**	***p*-value**
**Age (years)>55**	97 (51.1%)	72.1 ± 5.5	0.449
**Male gender**	158 (83.2%)	80.2 ± 5.0	0.676
**Liver cirrhosis (+)**	96 (50.5%)	76.7 ± 5.9	0.657
**LC3**			0.039
(**-**)	150 (78.9%)	74.9 ± 5.2	
(**+**)	40 (21.1%)	92.2 ± 8.3	
**LC3 stain pattern**			0.805
No stain	8 (4.2%)	48.1 ± 9.8	
Diffuse cytoplasmic	131 (68.9%)	77.0 ± 5.3	
Cytoplasmic/juxtanuclear	36 (18.9%)	77.6 ± 11.6	
Stone like structure	15 (7.9%)	78.9 ± 11.2	
**Child Pugh class**			0.028
A	188 (98.9%)	79.9 ± 4.6	
B	2 (1.1%)	28.9 ± 28.0	
**BCLC stage**			<0.001
0, A	74 (38.9%)	101.6 ± 7.0	
B, C	116 (61.1%)	65.7 ± 5.5	
**TNM stage**			<0.001
I-II	110 (57.9%)	93.3 ± 6.0	
III	80 (42.1%)	59.1 ± 6.4	
**Edmondson-Steiner Grades**			0.008
I-II	36 (18.9%)	104.3 ± 9.5	
III-IV	154 (81.1%)	73.9 ± 5.0	
**Tumor size**			<0.001
≤5cm	114 (60%)	91.2 ± 5.8	
>5cm	76 (40%)	61.1 ± 6.6	
**Serum AST**			0.021
≤50	135 (71.1%)	86.4 ± 5.5	
>50	55 (28.9%)	61.7 ± 6.8	
**Serum ALT**			0.738
≤50	138 (72.6%)	80.3 ± 5.2	
>50	52 (27.4%)	62.9 ± 5.3	
**Alpha-fetoprotein >200**	56 (29.5%)	72.7 ± 8.4	0.201

**Table 3:** Multivariate analysis for overall survival of patients with HCC resection.

**Variables**	**Hazard ratio**	**95% C.I.**	***p*-value**
**LC3(+)**	0.42	0.22 - 0.80	0.009
**BCLC stage (B, C)**	2.14	1.06 - 4.32	0.035
**Edmondson-Steiner Grades III-IV**	3.42	1.55 - 7.57	0.002
**Serum AST > 50**	1.82	1.11 - 2.98	0.016
**TNM stage III**	1.85	0.83–4.12	0.131
**Child Pugh class B**	2.13	0.49–9.12	0.308
**Tumor size > 5cm**	1.11	0.49–2.46	0.799

### Factors related to time to recurrence (TTR)

**Figure 3:**
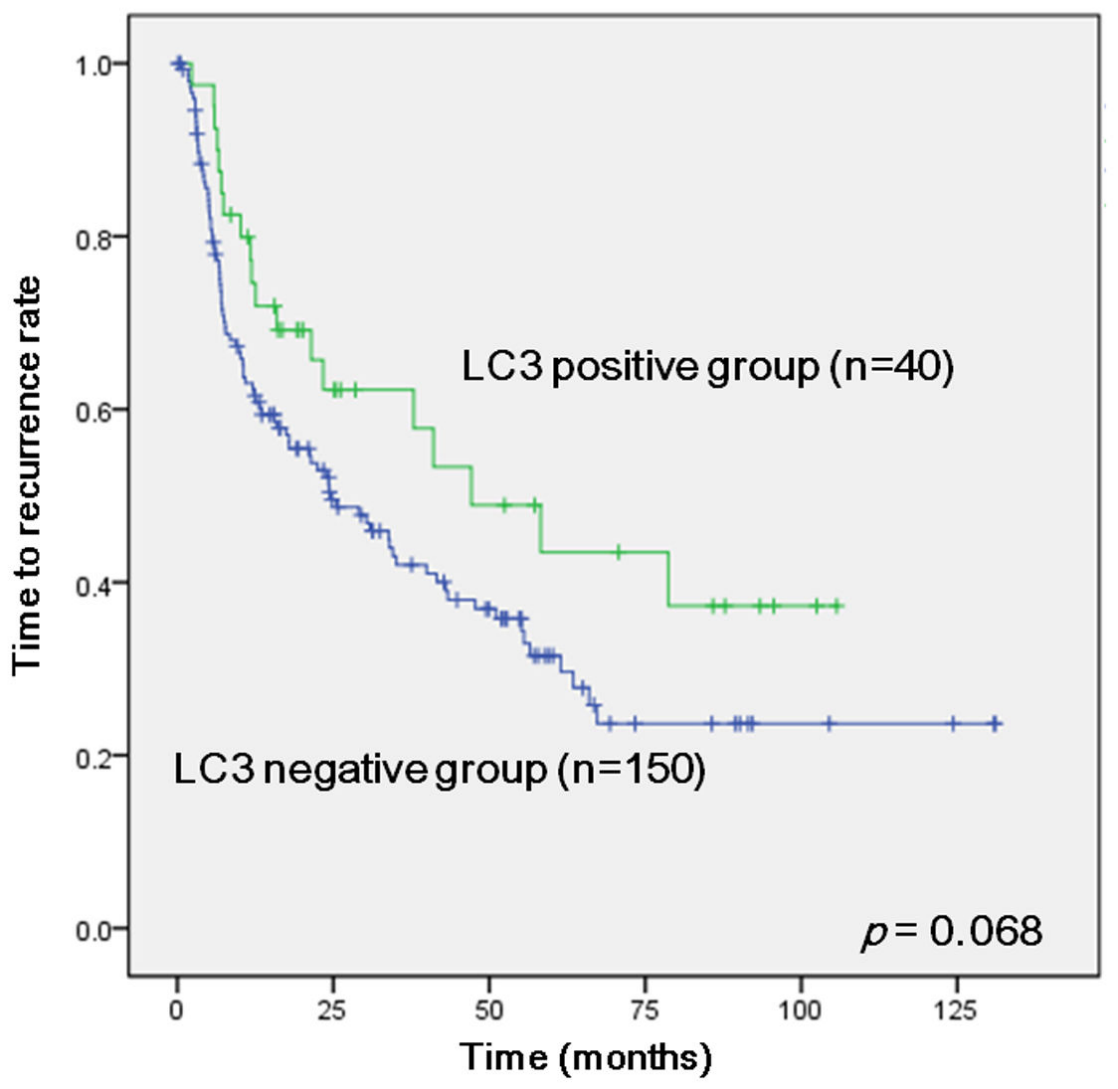
Kaplan–Meier curves of time to recurrence in hepatocellular carcinoma patients. LC3 positive group (n = 40) and LC3 negative group (n = 150).

HCC recurred in 110 (57.9%) patients, and the median recurrence time was 17.7 (range 0.2–136.0) months. Twelve (6.3%) patients’ cause of death was not HCC recurrence-related. We investigated factors related to TTR using both univariate and multivariate analyses. In the univariate analyses, BCLC stages 0 and A (*p*=0.001), TNM stages I and II (*p*<0.001), Edmondson–Steiner grades I and II (*p*=0.048), tumors ≤ 5 cm in size (*p*=0.004), and low serum AST and ALT (*p*=0.013 and *p*=0.046) were associated with longer TTR. The LC3 expression did not show significance, but tended to be associated with longer TTR (*p*=0.068, Figure 3) (Table 4). However, expressions of Beclin-1, GRP78 and CHOP were not associated with longer TTR (Table S2). Finally, positive LC3 expression (HR, 0.54; 95% CI, 0.33–0.90; *p*=0.017) and Edmondson–Steiner Grades III and IV (HR, 2.02; 95% CI, 1.16–3.52; *p*=0.013) were correlated with TTR in the multivariate analysis (Table 5). The three patterns of autophagic reactivity (diffuse cytoplasmic, cytoplasmic/juxtanuclear, and “stone-like” patterns) affected neither OS nor TTR (Table 2, 4). TTR was also analyzed according to HCC stage and LC3 expression, but LC3 expression did not differ significantly at any stage. Other markers except LC3 were not significantly associated with OS and TTR in HCC (Table S1 and S2).

**Table 4:** Univariate analyses for time to recurrence of patients with HCC resection.

**Variables**	**Number of Patients (n=190)**	**Mean survival (Mo)**	***p*-value**
**Age (years)>55**	97 (51.1%)	42.5 ± 4.1	0.853
**Male gender**	158 (83.2%)	51.4 ± 4.7	0.916
**Liver cirrhosis (+)**	96 (50.5%)	43.1 ± 4.2	0.817
**LC3**			0.068
(-)	150 (78.9%)	48.2 ± 4.8	
(+)	40 (21.1%)	57.3 ± 7.6	
**LC3 stain pattern**			0.698
No stain	8 (4.2%)	24.0 ± 7.9	
Diffuse cytoplasmic	131 (68.9%)	52.8 ± 5.4	
Cytoplasmic/juxtanuclear	36 (18.9%)	48.1 ± 8.6	
Stone like structure	15 (7.9%)	50.9 ± 11.6	
**Child Pugh class**			0.801
A	188 (98.9%)	50.0 ± 4.8	
B	2 (1.1%)	57.8 ± 8.6	
**BCLC stage**			0.001
0, A	74 (38.9%)	65.3 ± 7.2	
B, C	116 (61.1%)	43.1 ± 5.3	
**TNM stage**			< 0.001
I-II	110 (57.9%)	61.0 ± 5.8	
III	80 (42.1%)	40.0 ± 6.4	
**Edmondson-Steiner Grades**			0.048
I-II	36 (18.9%)	73.1 ± 11.1	
III-IV	154 (81.1%)	47.7 ± 4.6	
**Tumor size**			0.004
≤5cm	114 (60%)	58.4 ± 5.6	
>5cm	76 (40%)	41.5 ± 6.5	
**Serum AST**			0.013
≤50	135 (71.1%)	59.8 ± 5.4	
>50	55 (28.9%)	34.4 ± 5.8	
**Serum ALT**			0.046
≤50	138 (72.6%)	58.7 ± 5.3	
>50	52 (27.4%)	30.0 ± 4.2	
**Alpha-fetoprotein >200**	56 (29.5%)	56.4 ± 8.5	0.830

**Table 5:** Multivariate analysis for time to recurrence in patients with HCC resection.

**Variables**	**Hazard ratio**	**95% C.I.**	***p*-value**
**LC3(+)**	0.54	0.33–0.90	0.017
**Edmondson-Steiner Grades III-IV**	2.02	1.16–3.52	0.013
**BCLC stage (B, C)**	1.67	0.97–2.88	0.062
**TNM stage (III)**	1.72	0.92–3.21	0.086
**Tumor size > 5cm**	1.17	0.62–2.21	0.615
**Serum AST > 50**	1.39	0.85–2.28	0.179
**Serum ALT > 50**	1.52	0.92–2.53	0.100

## Discussion

 Autophagy is an evolutionarily conserved catabolic process that degrades cytoplasmic proteins and organelles via lysosomes. It includes three major intracellular pathways in eukaryotic cells: macroautophagy (the best-known pathway), microautophagy, and chaperone-mediated autophagy [21]. Autophagy in the liver plays important roles, including the balance of nutrients and energy, the removal of misfolded proteins, and the turnover of ER stress. Therefore, disturbances in autophagy function in the liver might have critical effects on liver physiology and liver disease, such as metabolic disturbance, cell death, inflammation, cirrhosis, and HCC [22]. Among the diverse types of cancer, the role of autophagy in HCC has been relatively well explored [23]; however, the role of autophagy as a prognostic parameter in HCC remains unclear.

 The expression of Beclin-1 and LC3, which are widely used markers in mammalian autophagy, has been investigated in a variety of tumor types [20,24-34]. Decreased Beclin-1 expression is frequently seen in malignant cells such as breast, esophageal, gastric, cervical, and stage III colon cancer cells, with a poor prognosis [24,25,29,31,33]. By contrast, other authors have demonstrated that increased Beclin-1 expression is associated with relatively poor clinical results in pancreatic and nasopharyngeal cancer [35]. Increased LC3 expression typically has unfavorable results in lung and pancreatic cancers and melanoma [30,34], while decreased LC3 expression has been correlated with tumorigenesis and a poor prognosis in breast cancer and early stage cervical squamous cell carcinoma [20,33]. Combined, these results imply that autophagy-related markers play different roles in different cancer types and stages. Therefore, we postulated that autophagy-related proteins play different roles in HCC progression, and the prognosis of HCC might vary according to tumor stage.

 Recently, many studies have demonstrated that ER stress and the UPR are strongly induced in diverse tumors and are closely associated with tumorigenesis [4]. However, our results showed that CHOP and GRP78, markers of ER stress, were not significantly correlated with other clinical characteristics and prognosis.

 Our study has demonstrated the following in HCC: (i) LC3 expression was significantly associated with longer OS and TTR regardless of tumor stage and liver function; (ii) in particular, LC3 expression was correlated with longer survival in advanced TNM stages (III) and Edmondson–Steiner grades (III, IV); and (iii) ER stress-related proteins were not significantly correlated with autophagy-related proteins. As mentioned above, the correlation between LC3 and tumor progression can be positive or negative, although the former is predominant. In our study, LC3 expression was significantly correlated with good clinical results in HCC, not only in terms of higher OS, but also in higher TTR. This implies that LC3 is an important prognostic factor in patients with curatively resected HCC. The reason that increased LC3 expression was associated with a good prognosis in HCC was not clear. According to the current understanding, autophagy might serve both suppressive and promoting roles in hepatocarcinogenesis. As a tumor-suppressing mechanism, defective autophagy contributes to tumor progression and malignant transformation. However, autophagy promotes the growth and survival of cancer cells in response to cellular stress, and then facilitates tumorigenesis and leads to resistance to therapy [23]. In addition, complex mechanisms seem to be involved in the development of HCC, and alternative pathways independent of LC3 control might be involved. Furthermore, the mechanisns of markers might differ according to the organ, cancer cell type, or stage [36]. Therefore, further studies of molecular signals for each cancer type and stage are needed.

 LC3 is considered a hallmark of autophagy that exists as soluble LC3-I and lipidated LC3-II [37]. LC3-I is converted into LC3-II and degraded after autophagosomes fuse with lysosomes [38]. Therefore, LC3-II is a reliable marker of LC3 [13], and the quantity of LC3-II is associated with the number of autophagosomes, which predicts autophagic activity. However, increased LC3 expression cannot accurately reflect increased autophagic activity; it can also indicate a decrease in autophagic function as a result of a block in fusion after increases in the numbers of autophagosomes [3]. True autophagic activity can be estimated by measuring the number of LC3-containing vesicles and autophagy flux using other techniques, because IHC

staining for LC3 cannot distinguish LC3-II from LC3-I. 

A recent study reported that “stone-like” LC3 expression in HCC is an independent predictor in HCC [39]. Similarly, Karpathiou et al. revealed that high stone-like structure counts were associated with a poor prognosis in lung cancer [34]. However, the three patterns of autophagic activity did not affect prognosis in our study. This finding might be due to the relatively small number of patients who had a “stone-like” LC3 expression, which prevented the data from reaching statistical significance.

Notably, in the baseline characteristics, LC3 expression was not related to liver function, tumor stage, or tumor cell differentiation, but it was related to the OS and TTR. We also found that the OS of subgroups with LC3 expression was longer than that of their counterparts without LC3 expression, especially in advanced HCC.

We targeted patients who underwent complete hepatic resections. As a result, all but two of the enrolled patients were C–P class A, which indicates favorable liver function. In this way, the data analysis reflects the real impact of tumor biology on clinical outcomes, without the unexpected effects of different therapies for HCC and the patients’ baseline parameters. These findings suggest that LC3 has prognostic value for HCC that is not influenced by other clinical parameters, particularly in patients with advanced HCC. 

Generally, advanced stages worse than BCLC stage A and TNM stage II are unlikely to indicate resection. In recent studies, however, resection of huge HCCs (≥ 10 cm) and advanced HCCs resulted in lower recurrence and longer survival rates than in patients receiving non-surgical treatments due to advances in techniques [40-42]. Our study included large and advanced HCC patients, and can be understood in the same context. Therefore, more aggressive treatment, such as adjuvant chemotherapy or radiotherapy, and short-term follow-up should be considered in advanced-stage HCC patients with LC3 expression after hepatic resection.

Several studies have reported that ER stress is a potent inducer of autophagy, and autophagy is also important for sustaining ER homeostasis [13,43]. In our study, however, ER stress- and autophagy-related markers were not associated with clinical outcomes, and there were no significant correlations among the markers.

This study was limited by being a single-center study with a retrospective design, which could lead to selection bias. Moreover, we used only IHC staining to identify LC3; therefore, we cannot distinguish LC3-II from LC3-I, and autophagy flux was not investigated. Finally, in our study, there was no difference in the prognosis of HCC according to LC3 pattern, likely because few stone-like LC3 cases were included. Hence, our findings should be considered within the context of these limitations. 

In conclusion, our study showed that LC3 expression was correlated with a good prognosis regardless of tumor stage or liver function. This suggests that LC3 is a useful marker of HCC that is not influenced by other clinical parameters. Furthermore, LC3 was correlated with longer overall survival, particularly in patients with advanced HCC. However, as mentioned above, increased LC3 expression in IHC staining cannot accurately reflect autophagic flux, it is hard to apply to therapeutic strategies yet. LC3 expression might serve as a strong prognostic factor and provide a therapeutic strategy for patients with advanced HCC after curative resections. Therefore, we should more pay attention to these patients including early induction of target therapy or meticulous surveillance. No autophagy-related markers were identified as being associated with ER stress-related markers. Further studies with more subjects are needed to clarify these results, and will provide new insights into the management of HCC. 

## Supporting Information

Figure S1 **Beclin-1 expression by immunohistochemistry in resected hepatocellular carcinoma (HCC).** Representative images of areas according to the proportion of positive cells (A–D) and intensity of staining (E–H). (A) none, (B) < 10%, (C) 10–50%, (D) > 50%; and staining (E) absent, (F) weak, (G) moderate, (H) strong. (LC3 stain, ×100) (upper panel, X 200; lower panel, X 400).
(TIF)

Figure S2 **CHOP expression by immunohistochemistry in resected hepatocellular carcinoma (HCC).** Representative images of areas according to the proportion of positive cells (A–D) and intensity of staining (E–H). (A) none, (B) < 10%, (C) 10–50%, (D) > 50%; and staining (E) absent, (F) weak, (G) moderate, (H) strong. (LC3 stain, ×100) (upper panel, X 200; lower panel, X 400).
(TIF)

Figure S3 **GRP78 expression by immunohistochemistry in resected hepatocellular carcinoma (HCC).** Representative images of areas according to the proportion of positive cells (A–D) and intensity of staining (E–H). (A) none, (B) < 10%, (C) 10–50%, (D) > 50%; and staining (E) absent, (F) weak, (G) moderate, (H) strong. (LC3 stain, ×100) (upper panel, X 200; lower panel, X 400).
(TIF)

Table S1 **Univariate analyses for overall survival of patients with HCC resection.**
(DOCX)

Table S2 **Univariate analyses for progression free survival of patients with HCC resection.**
(DOCX)
