# Correction: The Autophagy-Related Marker LC3 Can Predict Prognosis in Human Hepatocellular Carcinoma

**DOI:** 10.1371/annotation/154eb7f6-687c-4416-afc6-006658035c57

**Published:** 2013-12-20

**Authors:** Yoo Jin Lee, Yu Jin Hah, Yu Na Kang, Koo Jeong Kang, Jae Seok Hwang, Woo Jin Chung, Kwang Bum Cho, Kyung Sik Park, Eun Soo Kim, Hye-Young Seo, Mi-Kyung Kim, Keun-Gyu Park, Byoung Kuk Jang

The name of the second author was spelled incorrectly. The correct name is: Yu Jin Hah.

In addition, the name of the third author was incorrectly represented in the Citation. The correct Citation is: Lee YJ, Hah YJ, Kang YN, Kang KJ, Hwang JS, et al. (2013) The Autophagy-Related Marker LC3 Can Predict Prognosis in Human Hepatocellular Carcinoma. PLoS ONE 8(11): e81540. doi:10.1371/journal.pone.0081540 

