# Supplementary figures and images for: The Autophagy-Related Marker LC3 Can Predict Prognosis in Human Hepatocellular Carcinoma
Source: PLoS One. 2013 Nov 25;8(11):e81540. doi: 10.1371/journal.pone.0081540 (PMC3839913; doi:10.1371/journal.pone.0081540)

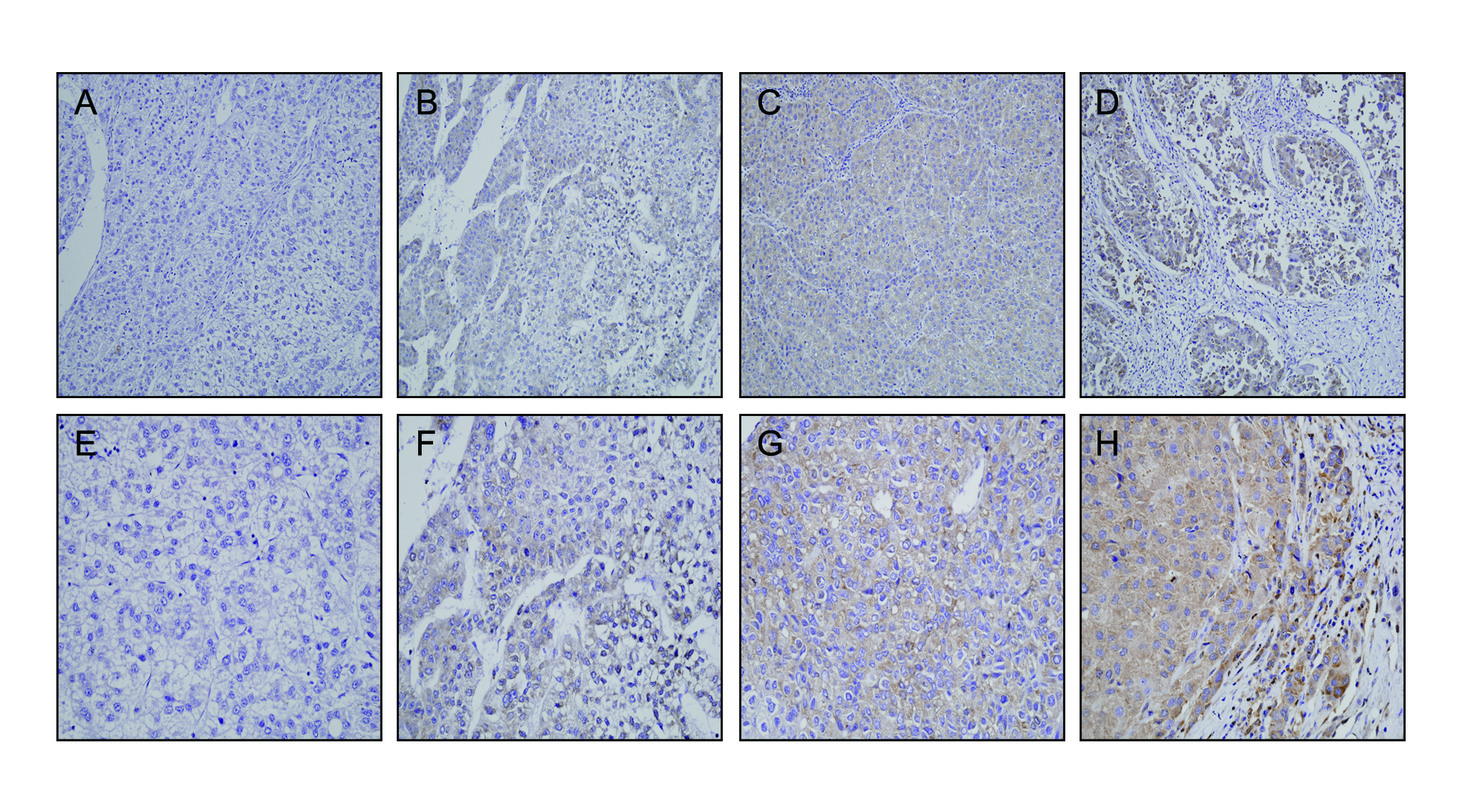

Supplement: Figure S1 — Beclin-1 expression by immunohistochemistry in resected hepatocellular carcinoma (HCC). Representative images of areas according to the proportion of positive cells (A–D) and intensity of staining (E–H). (A) none, (B) < 10%, (C) 10–50%, (D) > 50%; and staining (E) absent, (F) weak, (G) moderate, (H) strong. (LC3 stain, ×100) (upper panel, X 200; lower panel, X 400). (TIF) [file pone.0081540.s001.tif]

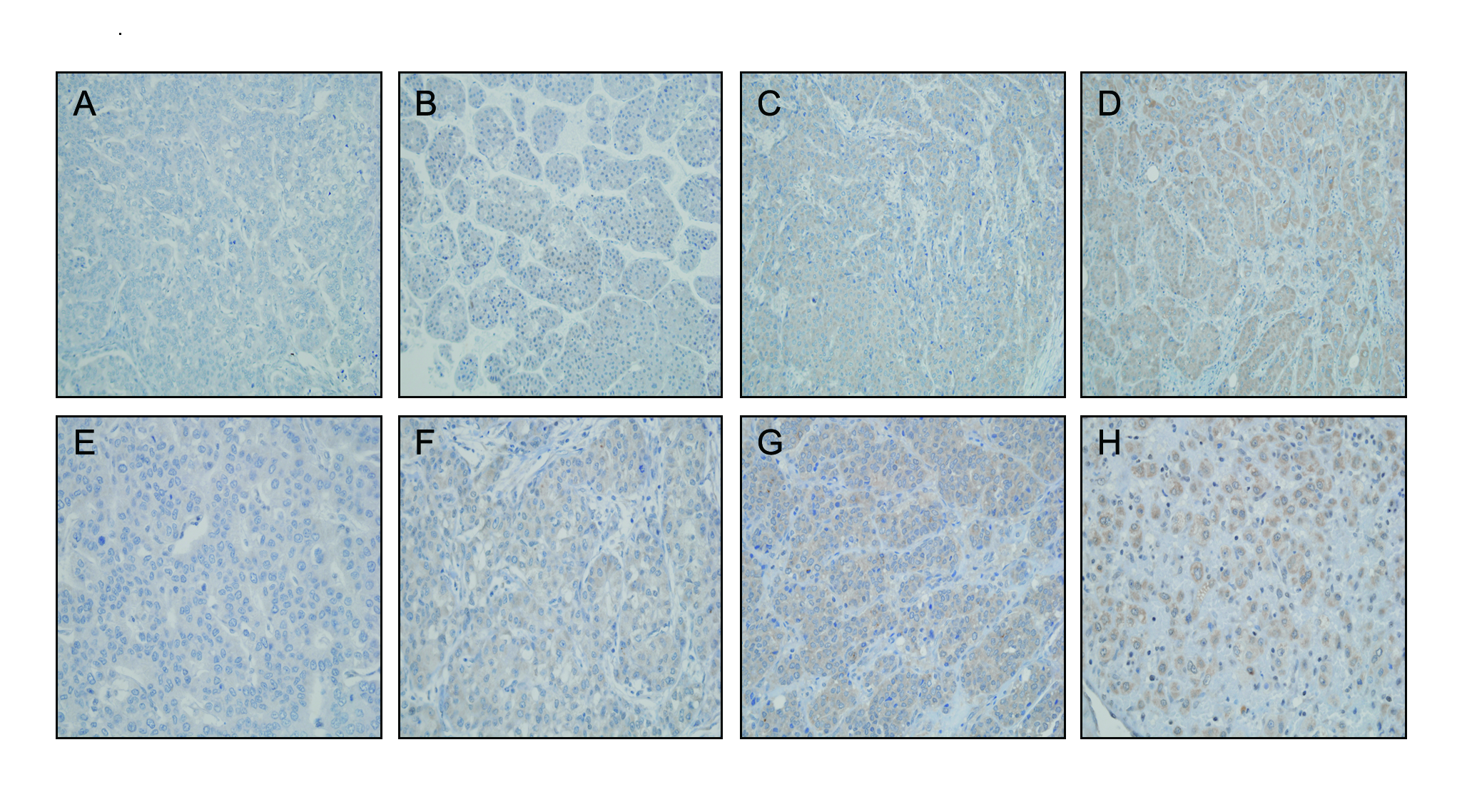

Supplement: Figure S2 — CHOP expression by immunohistochemistry in resected hepatocellular carcinoma (HCC). Representative images of areas according to the proportion of positive cells (A–D) and intensity of staining (E–H). (A) none, (B) < 10%, (C) 10–50%, (D) > 50%; and staining (E) absent, (F) weak, (G) moderate, (H) strong. (LC3 stain, ×100) (upper panel, X 200; lower panel, X 400). (TIF) [file pone.0081540.s002.tif]

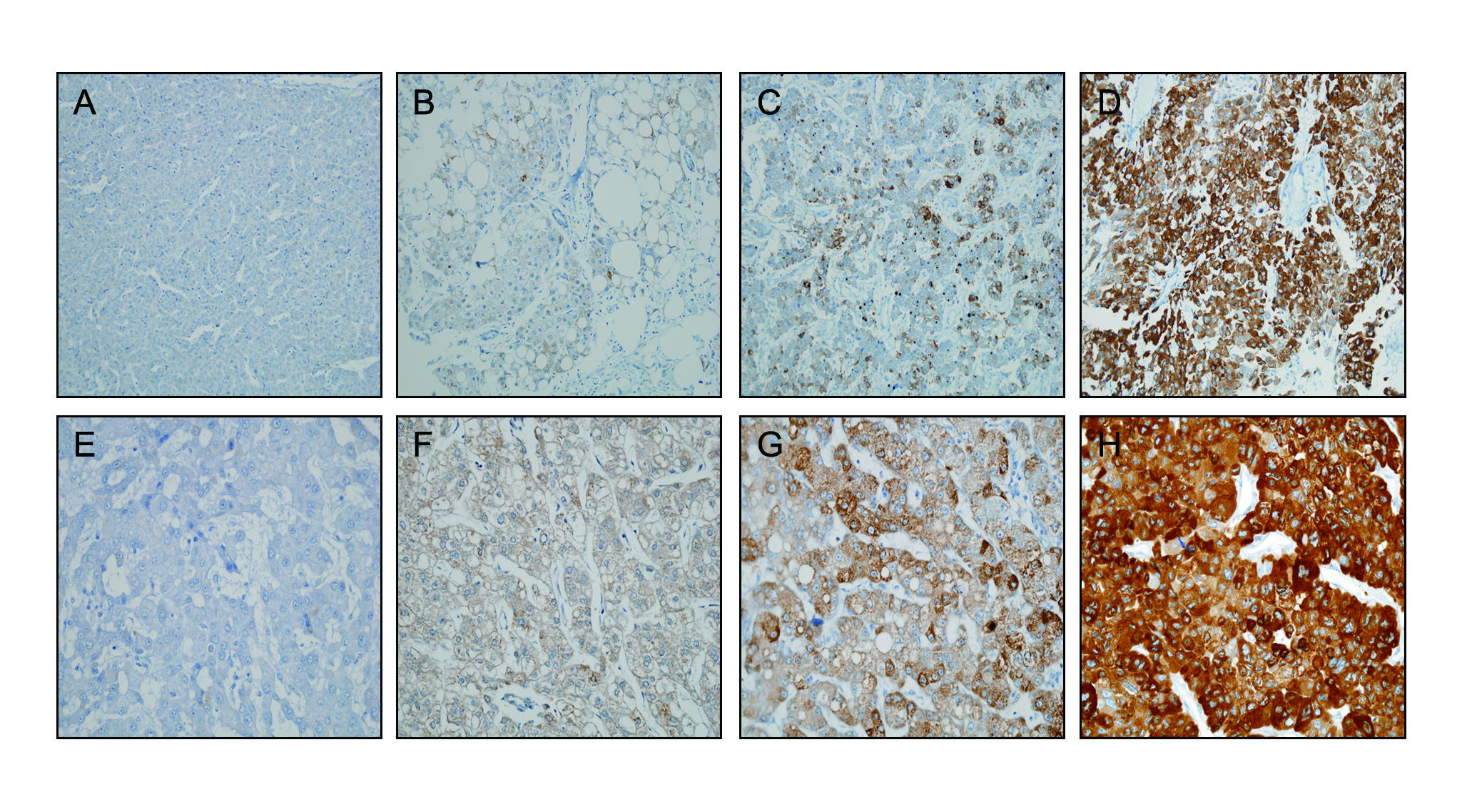

Supplement: Figure S3 — GRP78 expression by immunohistochemistry in resected hepatocellular carcinoma (HCC). Representative images of areas according to the proportion of positive cells (A–D) and intensity of staining (E–H). (A) none, (B) < 10%, (C) 10–50%, (D) > 50%; and staining (E) absent, (F) weak, (G) moderate, (H) strong. (LC3 stain, ×100) (upper panel, X 200; lower panel, X 400). (TIF) [file pone.0081540.s003.tif]
