# Supplementary material for: The Autophagy-Related Marker LC3 Can Predict Prognosis in Human Hepatocellular Carcinoma
Source: PLoS One. 2013 Nov 25;8(11):e81540. doi: 10.1371/journal.pone.0081540 (PMC3839913; doi:10.1371/journal.pone.0081540)
Supplement: Table S1 — Univariate analyses for overall survival of patients with HCC resection. (DOCX) [file pone.0081540.s004.docx]

**Table S1. Univariate analyses for overall survival of patients with HCC resection**

| **Variables** | **No. of Patients (n=190)** | **Mean survival (Mo)** | ***p*-value** |
| --- | --- | --- | --- |
| Beclin-1 |  |  | 0.842 |
| (-) | 179 (94.2%) | 79.6 ± 4.7 |  |
| (+) | 11 (5.8%) | 58.2 + 10.2 |  |
| GRP78 |  |  | 0.342 |
| (-) | 130 (68.4%) | 76.1 ± 5.2 |  |
| (+) | 60 (31.6%) | 82.7 ± 7.7 |  |
| CHOP |  |  | 0.061 |
| (-) | 184(96.8%) | 80.6 ± 4.6 |  |
| (+) | 6 (3.2%) | 48.3 ± 14.4 |  |
