# Supplementary material for: The Autophagy-Related Marker LC3 Can Predict Prognosis in Human Hepatocellular Carcinoma
Source: PLoS One. 2013 Nov 25;8(11):e81540. doi: 10.1371/journal.pone.0081540 (PMC3839913; doi:10.1371/journal.pone.0081540)
Supplement: Table S2 — Univariate analyses for progression free survival of patients with HCC resection. (DOCX) [file pone.0081540.s005.docx]

**Table S2. Univariate analyses for progression free survival of patients with HCC resection**

| **Variables** | **Number of Patients (n=190)** | **Mean survival (Mo)** | ***p*-value** |
| --- | --- | --- | --- |
| Beclin-1 |  |  | 0.256 |
| (-) | 179 (94.2%) | 53.3 ± 4.5 |  |
| (+) | 11 (5.8%) | 28.6 + 9.5 |  |
| GRP78 |  |  | 0.161 |
| (-) | 130 (68.4%) | 55.9 ± 5.3 |  |
| (+) | 60 (31.6%) | 34.7 ± 4.8 |  |
| CHOP |  |  | 0.791 |
| (-) | 184(96.8%) | 52.5 ± 4.4 |  |
| (+) | 6 (3.2%) | 51.8 ± 19.0 |  |
